# Oral tongue cancer gene expression profiling: Identification of novel potential prognosticators by oligonucleotide microarray analysis

**DOI:** 10.1186/1471-2407-9-11

**Published:** 2009-01-12

**Authors:** Cherry L Estilo, Pornchai O-charoenrat, Simon Talbot, Nicholas D Socci, Diane L Carlson, Ronald Ghossein, Tijaana Williams, Yoshihiro Yonekawa, Yegnanarayana Ramanathan, Jay O Boyle, Dennis H Kraus, Snehal Patel, Ashok R Shaha, Richard J Wong, Joseph M Huryn, Jatin P Shah, Bhuvanesh Singh

**Affiliations:** 1 Dental Service, Department of Surgery, Memorial Sloan-Kettering Cancer Center, NY, USA; 2 Division of Head and Neck Surgery, Department of Surgery, Siriraj Hospital Medical School, Bangkok, Thailand; 3 Laboratory of Epithelial Cancer Biology, Memorial Sloan-Kettering Cancer Center, NY, USA; 4 Department of Biostatistics, Memorial Sloan-Kettering Cancer Center, NY, USA; 5 Department of Pathology, Memorial Sloan-Kettering Cancer Center, NY, USA; 6 Head and Neck Service, Department of Surgery, Memorial Sloan-Kettering Cancer Center, NY, USA

## Abstract

**Background:**

The present study is aimed at identifying potential candidate genes as prognostic markers in human oral tongue squamous cell carcinoma (SCC) by large scale gene expression profiling.

**Methods:**

The gene expression profile of patients (n=37) with oral tongue SCC were analyzed using Affymetrix HG_U95Av2 high-density oligonucleotide arrays. Patients (n=20) from which there were available tumor and matched normal mucosa were grouped into stage (early vs. late) and nodal disease (node positive vs. node negative) subgroups and genes differentially expressed in tumor vs. normal and between the subgroups were identified. Three genes, *GLUT3*, *HSAL2*, and *PACE4*, were selected for their potential biological significance in a larger cohort of 49 patients via quantitative real-time RT-PCR.

**Results:**

Hierarchical clustering analyses failed to show significant segregation of patients. In patients (n=20) with available tumor and matched normal mucosa, 77 genes were found to be differentially expressed (P< 0.05) in the tongue tumor samples compared to their matched normal controls. Among the 45 over-expressed genes, *MMP-1* encoding interstitial collagenase showed the highest level of increase (average: 34.18 folds). Using the criterion of two-fold or greater as overexpression, 30.6%, 24.5% and 26.5% of patients showed high levels of *GLUT3*, *HSAL2* and *PACE4*, respectively. Univariate analyses demonstrated that *GLUT3* over-expression correlated with depth of invasion (P<0.0001), tumor size (P=0.024), pathological stage (P=0.009) and recurrence (P=0.038).  *HSAL2* was positively associated with depth of invasion (P=0.015) and advanced T stage (P=0.047).   In survival studies, only *GLUT3* showed a prognostic value with disease-free (P=0.049), relapse-free (P=0.002) and overall survival (P=0.003). *PACE4* mRNA expression failed to show correlation with any of the relevant parameters.

**Conclusion:**

The characterization of genes identified to be significant predictors of prognosis by oligonucleotide microarray and further validation by real-time RT-PCR offers a powerful strategy for identification of novel targets for prognostication and treatment of oral tongue carcinoma.

## Background

Cancer arising from the oral cavity accounts for approximately 1.6% of all cancers diagnosed in the United States with an incidence of 22,000 new cases per year [1]. Despite the advances in multimodality treatment, the overall prognosis for patients with oral squamous cell carcinoma (SCC) has remained unchanged in the past three decades. Furthermore, variability in the clinical course of patients with oral SCC remains unexplained and conventional clinicopathological parameters fail to answer all questions. Identification of novel prognostic factors may allow a rational selection of the most appropriate therapeutic options for individual patients. The cellular and molecular heterogeneity of oral SCC and the large number of genes potentially involved in oral carcinogenesis and progression emphasize the importance of studying multiple gene alterations on a global scale. Gene expression profiling by high-throughput technologies have proven to be valuable tools for prognostication of outcome and progression in human malignancies including head and neck cancer [2-10]. These technologies permit us to classify individual cancers and enhance our understanding of molecular cancer pathogenesis.

There are several distinct subsites within the oral cavity cancer including buccal mucosa, oral tongue, floor of mouth, gingiva, retromolar trigone and hard palate. Since they differ in their biological and clinical behaviors, the present study focused on one subsite – the oral tongue. This study utilized high-density oligonucleotide array to generate a molecular portrait of oral tongue SCC and to explore the correlations between gene expression patterns and clinically relevant parameters. We performed hierarchical clustering analysis, analyzed gene expression profiles by comparing primary tumor and their matched normal mucosa and compared different patient groups based on lymph node status and tumor stage to identify clinically significant genes. Data from the microarray analysis were then validated by real-time RT-PCR. The present study is the first to demonstrate the ability of gene expression profiling to predict clinical outcome in one cancer subsite within the oral cavity.

## Methods

### Tumor Selection

Following guidelines established by the Institutional Review Board at Memorial Sloan-Kettering Cancer Center (MSKCC), fresh tissue samples were sequentially collected after obtaining written informed consent from 49 patients undergoing therapeutic surgical resection for SCC of the oral tongue at the Head and Neck Service, MSKCC from January 28, 1998 to January 2, 2002. Post-operative adjuvant treatment was given to selected patients following the institutional protocol. In each case, the portion of tumor was resected near the advancing edge of the tumor to avoid its necrotic center. After excision, the tissues were immediately snap-frozen and stored in liquid nitrogen until use. Histologically normal mucosae of the upper aerodigestive tract, resected 5 cm away from the tumor area, were obtained in all cases and used as controls. Tumors were staged according to the AJCC/UICC TNM classification 5^th ^edition [11]. "Node-positive cases" in this study refers to the presence of positive cervical nodes based on a histological diagnosis after a neck dissection, while the patients who experienced no metastasis for at least 12 months post-operatively were scored as "node-negative cases." The clinical and pathological characteristics of all patients analyzed in the study are summarized in Table 1.

**Table 1:** Clinicopathological characteristics of the patients in the study and validation groups

Parameter	Study (Array)		Validation
	
	All samples	TN paired	RT-PCR
	
	NUMBER %		
Total	37	20	49

Age (yrs)			

Median (Range)	57 (36–97)	62 (35–97)	59 (35–97)

Gender			

Male	22 (59.5)	12 (60)	26 (53)

Female	15 (40.5)	8 (40)	23 (47)

Therapy prior to surgery			

None	22 (59.5)	12 (60.0)	34 (69.4)

Yes	15 (40.5)	8 (40.0)	15 (30.6)

Smoking history			

Yes	19 (51.4)	11 (55.0)	30 (61.2)

No	18 (48.6)	9 (45.0)	19 (38.8)

Alcohol history			

Yes	14 (37.9)	8 (40.0)	20 (40.8)

No	23 (62.1)	12 (60.0)	26 (59.2)

Unknown	0	0	3

Histological grading			

Well-differentiated	6 (16.2)	3 (15)	9 (18)

Mod-differentiated	24 (64.9)	13 (65)	32 (65)

Poorly-differentiated	7 (18.9)	4 (20)	8 (16)

Unknown	0	0	0

Lymph node involvement (Pathologic)			

Negative	26 (70.2)	13 (65)	25 (51)

Positive	11 (29.8)	7 (35)	24 (49)

Unknown	0	0	0

TNM Stage (Pathologic)			

I	8 (21.6)	4 (20)	11 (22)

II	12 (32.4)	6 (30)	12 (25)

III	3 (8.1)	3 (15)	7 (14)

IV	14 (37.9)	7 (35)	19 (39)

Unknown	0	0	0

### Oligonucleotide microarray analysis

Tumor and normal tissues from 37 of the 49 patients were used for the oligonucleotide microarray analysis. Twenty (TN paired) of the 37 patients had primary tumor samples and matched normal mucosa available for analysis. Total RNA from snap-frozen tissue samples from the 37 patients was extracted with TRIsol™ reagent (Gibco BRL) following the manufacturer's protocol and re-purified by the RNAeasy Mini-spin column (Qiagen). Five to 10 μg of total RNA was reverse transcribed in the presence of an oligo dT-T7 primer. The cDNA was used for *in vitro *transcription amplification reaction in the presence of biotinylated nucleotides. Fifteen μg of labeled cRNA was fragmented and then hybridized against the Affymetrix HG_U95Av2 oligonucleotide arrays (Affymetrix, Santa Clara, CA). The arrays were scanned using a Hewlett Packard confocal laser scanner and analyzed using MicroArray Suite 5.0 (Affymetrix).

The microarray data have been deposited in NCBIs Gene Expression Omnibus (GEO) http://www.ncbi.nlm.nih.gov/geo/ and can be accessed through GEO Series accession number GSE13601.

### RNA preparation and real-time RT-PCR

RT-PCR of *GLUT3, HSAL2*, and *PACE4 *was performed on the larger cohort of 49 patients. Two μg of total RNA was reverse transcribed with MultiScribe™ Reverse Transcriptase (Applied Biosystems, Inc.). Gene specific primers were designed using the Primer3 Program. Sequences of PCR primer sets (in 5'-3' direction) were as follows: *GLUT3 *forward: TAGAAAGCCTGTTCCCCTCA, *GLUT3 *backward: GTGGCGGGATTACTTCAAAA; *HSAL2 *forward: CCCTCCTATTTCAGCCTCCT, *HSAL2 *backward: TCTTCAGTACCGGCACCTTC; *PACE4 *forward: CCTGTGTGACCCTCTGTCCT, *PACE4 *backward: GGTTCATCCACGCACTTTTT. The sequence of PCR primer sets for *18S rRNA *were previously described [12]. Quantification of transcripts was performed by the ICycler Detection System (Bio-Rad Laboratories) using SYBR green detection. The relative quantification of a target gene in comparison to a reference (*18S rRNA*) was performed as described [13]. Unless otherwise stated, each assay included duplicate reactions for each sample and was repeated twice.

### Statistical analysis

All correlation and outcome analysis was performed using the JMP statistical software package version 4.0.0 (SAS Institute, Inc.). Disease-free survival is defined as the time from surgery to the day of the first recurrence or death. Relapse-free survival was defined as time from surgery to the day of the first recurrence. Overall survival was defined as time from surgery to the day of death or last follow-up.

## Results and discussion

### Gene expression patterns in oral tongue SCC

We analyzed gene expression profiles in 20 patients with oral tongue SCC by comparison between primary tumor samples and their matched morphologically normal mucosa. Among 12,625 probe sets in the Affymetrix array, 77 probe sets had statistically significant difference (P < 0.05) between all tumors and their matched normal tissues. There were 60 probe sets representing 45 genes and 11 ESTs that were increased and 17 probe sets representing 9 genes and 8 ESTs that were decreased in tumors compared to normal controls. Table 2 lists the genes that were up-regulated or down-regulated along with the fold changes in gene expression in tumor compared to their normal counterparts. These include genes known to be relevant in oncogenesis such as cell proliferation, apoptosis, development, angiogenesis, invasion and metastasis as well as genes that have not been implicated in oral tongue carcinogenesis. Among the 45 over-expressed genes, *MMP-1 *encoding interstitial collagenase showed the highest level of increase (average: 34.18 fold). *MMP-7 *and *MMP-12 *were also found to be overexpressed. Matrix metalloproteinases (MMPs), a family of 23 human zinc-dependent extracellular endopeptidases involved in the degradation of extracellular matrix and basement membrane during tumor cell invasion, have been implicated in a number of different human tumors including head and neck SCC [14,15]. Not surprisingly, enhanced *MMP-1 *expression has been found to be associated with malignant progression as well as poor outcome in head and neck SCC [15-17]. Likewise, *MMP-7 *and *MMP-12 *have both been implicated in tumor aggressiveness in oral SCC [18]. Genes that have been shown to be involved in epithelial development and differentiation such as the cytokeratins KRT16 and KRT17 were found to be overexpessed. In their study investigating RNA from head and neck SCC and normal tissues, Villaret et al. (2000) found *KRT6 *and *KRT16 *to be the genes most commonly expressed [19]. Similarly, *KRT16 *has also been found to be highly expressed in squamous cell carcinoma of the skin [20]. In addition, those that play a role in angiogenesis, such as hypoxia-inducible factor (*HIF-1α*) and platelet-derived endothelial cell growth factor (*ECGF1*) were also overexpressed. Several transcripts were found to be significantly under-expressed or absent in tumor compared with matched normal tissues including those that encode for cell surface (CO-029), nuclear (ZAKI-4) and extracellular proteins (hSBP).

**Table 2:** Genes differentially expressed between oral tongue tumor and case-matched histologically normal mucosae (TN paired, n = 20)

Accession	Sequence identity (GenBank/EMBL)	Average log fold change
**Nuclear proteins (transcription factors, DNA processing enzymes)**		

AJ001381	Mutated allele of a myosin class I, myh-1c	5.24

D28364	Annexin II	4.61

M92383	Thymosin beta-10	3.75

U22431	Hypoxia-inducible factor 1 alpha (HIF-1 alpha)	3.47

M86400	Phospholipase A2	3.27

L19779	Histone H2A.2	2.99

U10860	Guanosine 5-monophosphate synthase	2.71

U12472, U21689	Glutathione S-transferase (GST phi)	2.70

J04173	Phosphoglycerate mutase (PGAM-B)	2.69

AL009179	Histone H2B	2.58

D13748	Eukaryotic initiation factor 4AI	2.57

S79639	Exostosin 1 (EXT1)	2.39

AL049650	snRNP (small nuclear ribonucleoprotein particle) protein B)	2.35

	Ras-Like Protein Tc4	2.27

D26599	Proteasome subunit HsC7-I	2.04

X82554	SPHAR gene for cyclin-related protein	-1.89

U40490	Human nicotinamide nucleotide transhydrogenase	-3.53

D83407	ZAKI-4 in human skin fibroblast	-5.31

**Cytokines, growth factors and receptors**		

NM001953, M63193	Platelet-derived endothelial cell growth factor (ECGF1)	15.89

M77349	Transforming growth factor-beta induced gene product (BIGH3)	6.17

U73377	p66shc (SHC)	2.37

M35252	Tumor associated antigen CO-029	-9.42

**Signaling molecules**		

AF054183	GTP binding protein	2.00

U78525	Eukaryotic translation initiation factor (eIF3)	1.89

**Metabolic enzymes, transporters, ion channels**		

AF042498	Rod photoreceptor CNG-channel beta subunit (RCNC2)	6.73

M94856	Fatty acid binding protein homologue (PA-FABP)	5.24

M91670	Ubiquitin carrier protein (E2-EPF)	3.52

J03626	UMP synthase	2.54

D50840	Ceramide glucosyltransferase	2.54

U89606	Pyridoxal kinase	2.41

**Accession**	**Sequence identity (GenBank/EMBL)**	**Average log fold change**

X52851	Cyclophilin	2.22

X97074	Clathrin-associated protein	2.07

S81003	L-UBC = ubiquitin conjugating enzyme	2.04

U09510	Glycyl-tRNA synthetase	1.96

D25547	PIMT isozyme I	1.87

AF052941	DAP-kinase related protein 1	-2.12

D16294	Mitochondrial 3-oxoacyl-CoA thiolase	-3.59

**Extracellular proteins**		

M13509	Skin collagenase (MMP1)	34.18

X07820	Stromelysin-2 (MMP7)	15.89

L10343	Elafin	9.95

L23808	Macrophage metalloelastase (MME, MMP12)	7.67

U29091	Human selenium-binding protein (hSBP)	-5.41

**Others**		

AB018342	KIAA0799	4.44

D21261	KIAA0120	2.59

AB014515	KIAA0615	2.42

AB007889	KIAA0429	2.09

AA586894	EST	14.57

AA010777	EST	14.12

L05424	EST	5.76

AI885852	EST	4.03

Z98946	EST	3.42

AC002073	EST	3.17

AF053356	EST	1.73

AB028994	KIAA1071	-6.08

D42047	KIAA0089	-4.68

AB007972	KIAA0503	-3.96

AF007153	EST	-5.35

AI674208	EST	-3.57

AL080059	EST	-3.12

AL049381	EST	-2.93

AW021542	EST	-2.60

### Hierarchical clustering analysis

**Figure 1:**
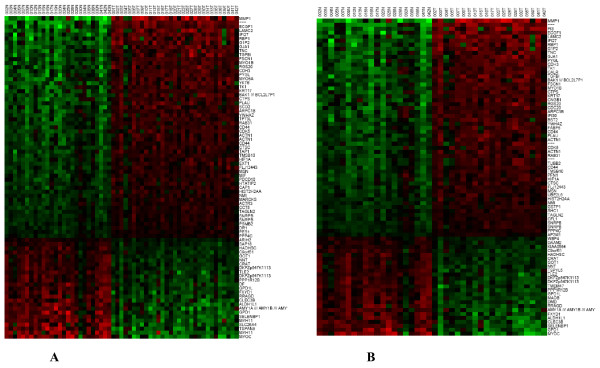
**Hierarchical clustering of the gene expression data for the TN cluster set, N = 37**. A; and the TN paired cluster set, N = 20. B. Approximately 12, 625 genes were clustered using the method described in the text. The genes shown represent the top 80 genes that were up-regulated (red) and down-regulated (green) in the sample sets. The normal tissue (N) and tumor tissue (T) are followed by their corresponding case numbers.

**Figure 2:**
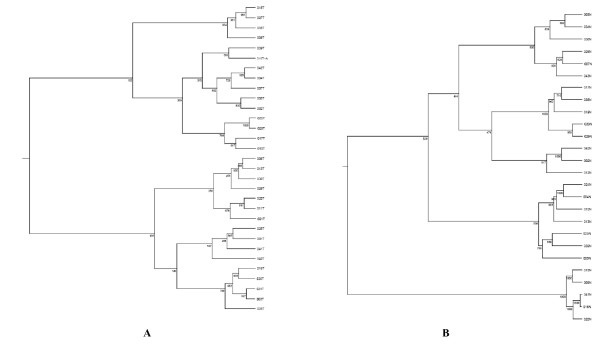
**Hierarchical clustering of tumor (T), N = 31**. A; and normal (N), N = 26. B cluster sets based on the complete panel of 12,000 genes. Horizontal distance represents the correlation of the samples to one another. The numbers at each node represent frequency of the shown pattern using 1,000 iteration bootstrap resampling.

Four separate clustering analyses were performed: (1) All tumor and normal samples (TN, n = 37), (2) the tumor and their matched normal samples (TN paired, n = 20); (3) the tumor samples by themselves (T, n = 31); and (4) the normal samples by themselves (N, n = 26). The data were clustered using the standard hierarchical method with ward linkage and using the Pearson correlation to determine the distance function. The distance between samples was dist = (1-p)/2 where p is the correlation coefficient. Before clustering, the data was filtered to remove genes that were scored Absent (A) by the MAS5.0 software in 75% or more of the samples as they are likely to be measuring noise in the system. To assess the robustness of the clustering results, a resampling method was used to create 1000 replica datasets by adding Guassian noise to each point. These 1000 data sets were individually clustered and then a consensus tree was built from them. The number at each node in the tree indicates how often that subtree appears in the 1000 replica trees. The higher the number, the more robust is the subtree. The samples from the TN cluster set clearly segregated the tumor from the normal samples (Figure 1A). Similarly, TN paired set separated the tumor samples from their matched normal counterparts (Figure 1B). The two clusters exhibit nearly identical patterns of gene expression changes. As shown on Table 2, the genes that were overexpressed included those involved in tumor invasion, epithelial development and angiogenesis. In contrast, however, clustering analysis failed to show significant segregation of patients based on expression profiling in both the T and N cluster sets possibly due to the heterogeneous nature of the samples as well as the relatively small number of samples in this study (Figure 2).

### Assessment of correlation with lymph node status, stage and outcome

**Figure 3:**
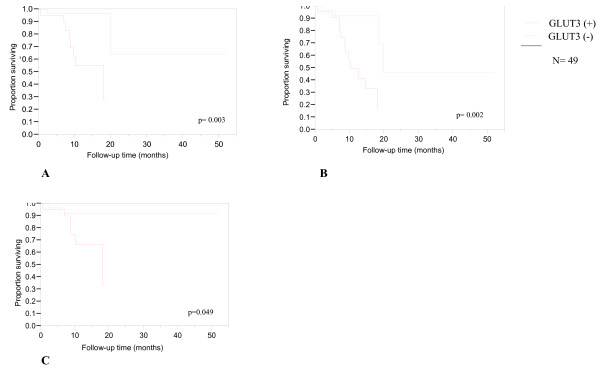
**Kaplan-Meier plot showing over-all survival**. A; relapse-free survival. B; and disease-free survival. C in patients demonstrating GLUT3 over-expression.

We grouped patients with pathologic stages I and II disease into an early-stage disease and grouped patients with pathologic stages III and IV disease into a late-stage disease category. Through statistical regression analysis, we identified genes whose expression differed in tumor versus normal mucosa and those whose expression was most different between the staging subgroups (Table 3A). The same analysis was performed to compare patients without cervical nodal metastasis (N0) to those with nodal disease (N1–N3) (Table 3B). We analyzed data from patients (n = 20) for which tumor and matched normal mucosae were available and not the larger subgroup of patients (n = 37) in order to obtain a more meaningful comparison of gene expression changes between tumor and normal mucosa. We selected three genes, *GLUT3, HSAL2 *and *PACE4 *for further analysis and validation in a larger cohort of 49 patients. We selected genes with known important roles in cellular functions and carcinogenesis and for which antibodies were available. We employed a two-step quantitative RT-PCR to validate expression changes identified by gene array analysis for the three selected genes in all 49 cases. We defined the cut-off value for over-expression as two-fold or greater relative to matched normal controls. Using these criteria, 30.6%, 24.5% and 26.5% of patients expressed high levels of *GLUT3, HSAL2 *and *PACE4*, respectively. We assessed the prognostic significance of expression of the selected genes and various clinicopathological parameters. Univariate analyses demonstrated that *GLUT3 *over-expression correlated with depth of invasion (P < 0.0001), tumor size (P = 0.024), pathological stage (P = 0.009) and recurrence (P = 0.038). *HSAL2 *was positively associated with depth of invasion (P = 0.015) and advanced T stage (P = 0.047). *PACE4 *expression failed to show correlation with clinicopathological parameters. Table 4 depicts the univariate analysis of *GLUT3, HSAL2 *and *PACE4 *expression and various clinicopathologic parameters. In survival studies, only *GLUT3 *showed a prognostic value with disease-free survival (P = 0.049), relapse-free survival (P = 0.002) and overall survival (P = 0.003) (Figure 3). Multivariate analysis with Cox's proportional hazards revealed that all parameters remained independent prognosticators in this group of patients.

**Table 3:** Differentially expressed genes associated with cervical lymph node and pathologic stage status

p-value	Number present	t-score	Accession: Definition/Description or EST name
0.001%	30	5.324	M20681: Human glucose transporter-like protein-III (Glu3)

0.006%	10	4.525	X71125: H. sapiens mRNA for glutamine cyclotransferase

0.011%	10	3.943	X51757: HAP70B Human heat-shock protein HSP70B gene

0.014%	16	4.519	AB028957: H. sapiens mRNA for KIAA1034 protein

0.025%	26	4.261	Protein Phosphatase inhibitor homolog

0.021%	30	4.568	Y12065: H. Sapiens mRNA for nucleolar protein hNop56

0.031%	29	4.272	AB015633: Homo sapiens mRNA for type II membrane protein

0.031%	8	4.517	D78579: Homo sapiens mRNA for neuron derived orphan receptor

0.031%	17	3.764	W25985: 17e6

0.044%	31	3.792	AF007140: Homo sapiens clone 23711 unknown mRNA

0.061%	31	3.659	X86163: H. sapiens mRNA for B2-bradykinin receptor

0.064%	31	3.706	A1935420: wo84c08

0.076%	8	3.188	AB020654: Homo sapiens mRNA for KIAA0847 protein

0.076%	7	3.246	L23959: HUMDP1A Homo sapiens E2F-related transcription factor (DP-1) mRNA

0.090%	31	2.808	AL09674: Homo sapiens mRNA: cDNA DKFZp58600223

0.090%	21	3.544	Z11697: Homo sapiens mRNA for HB 15

0.090%	22	3.813	X98834**: H. sapiens mRNA for zinc finger protein, Hsal2**

0.106%	16	3.720	M81750: H. sapiens myeloid cell nuclear differentiation antigen mRNA

0.106%	25	3.943	S68134: CREM = cyclic AMP-responsive element modulator beta isoform

0.125%	31	3.302	X98296: H. sapiens mRNA for ubiquitin hydrolase

0.147%	26	3.051	U49392: Human allograft inflammatory factor-1 (AIF-1) mRNA

0.193%	7	3.269	AL109669: Homo sapiens mRNA full length insert cDNA clone EUROIMAGE

0.195%	5	2.861	M31166: HUMTSG14A Human tumor necrosis factor-inducible (TSG-14) mRNA

**A**			

**p-value**	**Number present**	**t-score**	**Accession: Definition/Description or EST name**

0.016%	31	4.286	AA402538: Soares ovary tumor NbHOT Homo sapiens cDNA

0.035%	22	3.690	X98834**: H. sapiens mRNA for zinc finger protein, Hsal2**

0.035%	26	3.501	U49392: Human allograft inflammatory factory-1 (ALF-1) mRNA

0.043%	5	3.782	X62055: H. sapiens PTP1C mRNA for protein-tyrosine phosphate

**p-value**	**Number present**	**t-score**	**Accession: Definition/Description or EST name**

0.062%	11	3.514	AB023135: Homo sapiens mRNA for activation-inducible lymphocyte

			immunomediatory molecule AILIM

0.062%	31	3.698	A1828166: wk32h09

0.073%	31	3.761	U85773: Human phosphomannomutase (PMM2) mRNA

0.085%	6	3.501	S67970: ZNF75 = KRAB zinc finger [human, lung fibroblast, mRNA

0.104%	16	3.709	M81750: H. sapiens myeloid cell nuclear differentiation antigen mRNA

0.104%	31	3.824	AA121509:zk88c10.s1

**p-value**	**Number present**	**t-score**	**Accession: Definition/Description or EST name**

0.104%	21	3.023	Z11697: Homo sapiens mRNA for HB15

0.104%	31	3.952	D67031: Homo sapiens ADDL mRNA for adducin-like protein

0.123%	29	3.565	J02923: Human 65-kilodalton phosphoprotein (p65) mRNA

0.123%	21	3.271	L10717: Homo sapiens T cell-specified tyrosine kinase mRNA

0.145%	26	2.955	AL031228:dJ1033B10. 10/Membrane protein with histidine rich charge clusters

0.145%	24	3.512	AB011102: Homo sapiens mRNA for KIAA0530 protein

0.170%	25	2.843	M80482**: Human subtilisin-like protein (PACE4) mRNA**

0.199%	19	3.215	AF072099: Homo sapiens immunoglobulin-like transcript 3protein variant 1 gene

0.199%	28	3.319	J03037: Human carbonic anhydrase II mRNA

0.199%	31	2.979	D21261: Human mRNA for KIAA0120 gene

0.199%	31	3.542	L06797: HUMGPCR Human (clone L5) orphan G protein-coupled receptor mRNA

**B**			

The 20 patients were grouped into early-stage (Stage I and II disease) category and late-stage (Stage III and IV disease) category. A; Similarly, patients without cervical nodal metastasis (N0) were compared to those with nodal disease (N1–N3). B; Genes whose expression in tumor was different in normal mucosa and which were most different between the clinical stage and nodal disease subgroups were identified by statistical regression analysis (t test). The genes identified within the two subgroups are listed. Note that HSAL2 were identified in both subgroups. Only genes that were present in at least 5 samples were included.

**Table 4:** Univariate analysis of clinicopathologic parameters and gene expression

Parameter	GLUT3	HSAL2	PACE4
	p-value		

DOI	p < 0.0001	p = 0.015	NS

Tumor size	p = 0.024	NS	NS

pStage	p = 0.009	NS	NS

Advanced T stage	NS	p = 0.047	NS

Recurrence	p = 0.038	NS	NS

N^+^	NS	NS	NS

DOI = depth of invasion; pStage = pathologic stage; NS = not significant; N^+ ^= presence of lymph node metastasis

Malignant cells show an increased glucose uptake *in vitro *and *in vivo *[21,22]. This process is thought to be mediated by glucose transporters (GLUTs), the expression and activity of which is regulated by oncogenes, growth factors and cytokines [23,24]. Studies of *GLUT *genes in human cancers have shown over-expression of *GLUT1 *and *GLUT3 *in cancers of various sites including the head and neck [25-28]. Recent studies in laryngeal carcinoma demonstrated an association between *GLUT3 *protein levels and poorer outcome [29]. *HSAL2 *is a member of a gene family that encodes a group of putative transcription factors. Evidence from various studies suggests that the *HSAL *gene family is necessary for normal embryonic development and genetic alterations can lead to human congenital defects and cancer [30,31]. *HSAL2 *is thought to have a role as a tumor suppressor gene in ovarian cancer [32]. The present study suggests the potential role of *GLUT3 *and *HSAL2 *in oral tongue SCC. Proprotein convertases (PC) are a family of serine endoproteases that play important roles in regulating cell function by converting proproteins to biologically active molecules such as neuropeptides and polypeptide hormones, protein tyrosine phosphatases, growth factors and their receptors, and enzymes including MMPs. Numerous members of the PC family have been associated with invasion and proliferation in various cancers including head and neck, breast and lung cancers [33-35]. PCs are thought to activate certain substrates that may play a significant role in carcinogenesis. Among these substrates are MMPs which are known to be involved in the degradation of extracellular matrix, a key process in the initiation of tumor microinvasion into the connective tissue. PACE4, a member of the PC family, activates membrane type MMPs (MT-MMPs). Bassi et al. demonstrated that *PACE4 *expression results in enhanced susceptibility to carcinogenesis in *vivo *[35]. In the present study, *PACE4 *failed to show clinical significance when validated by real-time RT-PCR likely due to the small sample size and heterogeneous nature of the specimens.

There is growing literature on the use of microarray technology to examine genomewide genetic expression changes associated with head and neck SCC development and to identify biomarkers as it relates to response to therapy and clinical outcome [5-10]. However, there is discordance among the studies. Furthermore, the biomarkers often lack predictive power. Two important limitations of the studies to date are the heterogeneity in subsites analyzed and the small number of study samples. Our study as well as that of others addressed the former by focusing only on oral tongue SCC [36-38]. Carinci et al. analyzed gene expression in 9 specimens with dysplasia, 8 with oral tongue SCC with no metastasis, and 11 with metastatic oral tongue SCC. Several genes were identified as potential markers of oral tongue progression and metastasis [36]. Shimada and colleagues identified 16 genes that were upregulated in 4 oral tongue SCC specimens. One gene, *RabIA*, a member of the *Ras *oncogene, was chosen and validated at the RNA and protein levels [37]. In a study by Zhou et al., 25 primary oral tongue SCC were classified based on lymph node status and the presence of extracapsular spread. Among the genes that were shown to be associated with metastasis included *MMP-9 *[38].

Our findings show overexpression of numerous genes that have been previously shown in other DNA microarray studies to have a potential role in the development of head and neck carcinogenesis; these include *MMP-1 *and *KRT16*. Furthermore, we have identified and validated *GLUT3 *and *HSAL2 *to be potential prognosticator of head and neck SCC. Although the present study, like the others, is based on a relatively small number of patients and the findings do not allow us to draw definitive conclusion regarding their biological importance, it is the first to use large scale transcriptional profiling for predicting survival outcome in oral tongue SCC.

## Conclusion

The use of high-density oligonucleotide probe arrays to identify gene expression differences between oral tongue SCC and normal tissues provide powerful means to decode the molecular events involved in the genesis and progression of oral SCC. Although these initial findings will need to be validated in relationship to clinical parameters and outcome in larger patient cohorts, the characterization of genes identified to be significant predictors by oligonucleotide microarray analysis may provide novel targets for the prognostication and treatment of oral cavity cancer. Finally, a large multi-institutional study including specimens of uniform characteristics using independent techniques to verify gene expression at the RNA, DNA and protein levels will be vital in reaching our ultimate of goal of improving the care of head and neck cancer patients.

## Abbreviations

SCC: squamous cell carcinoma; CGH: comparative genomic hybridization; RT: reverse transcription; PCR: polymerase chain reaction; RR: risk ratio; CI: confidence intervals; EST: expressed sequence tag.

## Competing interests

The authors declare that they have no competing interests.

## Authors' contributions

CLE and PO performed all RNA extraction, RNA preparation and real-time RT-PCR experiments and correlation and outcome analysis. They also drafted the manuscript. ST, TW and YY collected clinical data on patients involved in the study. NS performed all statistical analysis of the microarray data. DLC and RG reviewed all the histologic specimens. JOB, DHK, SP, ARS, RJW, JMH and JPS assisted in the drafting of the manuscript. YR and BS supervised all work and aided in the drafting of the manuscript. All authors read and approved the final manuscript.

## Pre-publication history

The pre-publication history for this paper can be accessed here:

http://www.biomedcentral.com/1471-2407/9/11/prepub
